# Capsid-dependent lentiviral restrictions

**DOI:** 10.1128/jvi.00308-24

**Published:** 2024-03-18

**Authors:** Joy Twentyman, Michael Emerman, Molly Ohainle

**Affiliations:** 1 Division of Human Biology, Fred Hutchinson Cancer Center, Seattle, Washington, USA; 2 Department of Molecular and Cell Biology, Division of Immunology and Molecular Medicine, University of California Berkeley, Berkeley, California, USA; New York University Department of Microbiology, New York, New York, USA

**Keywords:** lentiviruses, capsid, human immunodeficiency virus, restriction factor

## Abstract

Host antiviral proteins inhibit primate lentiviruses and other retroviruses by targeting many features of the viral life cycle. The lentiviral capsid protein and the assembled viral core are known to be inhibited through multiple, directly acting antiviral proteins. Several phenotypes, including those known as *Lv1* through *Lv5*, have been described as cell type-specific blocks to infection against some but not all primate lentiviruses. Here we review important features of known capsid-targeting blocks to infection together with several blocks to infection for which the genes responsible for the inhibition still remain to be identified. We outline the features of these blocks as well as how current methodologies are now well suited to find these antiviral genes and solve these long-standing mysteries in the HIV and retrovirology fields.

## INTRODUCTION

The capsid (CA) protein of HIV-1 serves functions both early in the viral life cycle in targeting the viral core to the nucleus, as well as late in the viral life cycle by forming the core structural component of the virion (1, 2). The term “CA” will be used here to denote protein subunits. The term “capsid” will be used to refer to the assembled structure. CA is encoded by the viral *gag* gene, which is translated as a polyprotein and then cleaved into individual units including the CA protein that becomes the viral core after budding. Each viral core is composed of about 1,500 CA monomers which multimerize into approximately 250 hexamers and exactly 12 pentamers (1, 3). These hexamers and pentamers form the viral core that includes the viral RNA genome. In the early stages of the viral life cycle after entry into the host cell, the HIV-1 core is deposited in the cytoplasm and imported into the nucleus via the nuclear pore complex where reverse transcription is completed and integration into the host cell genome occurs (1). Host proteins bind to HIV-1 capsid both during its early phase in the viral life cycle and in its late phase. While some of these host proteins aid the virus in these processes, lentiviral capsids, including HIV, are also the target of host antiviral proteins (2). There are multiple phenotypes or blocks to lentiviral replication that have been characterized but for which a responsible host protein or proteins have not been fully or definitively identified, for example *Lv2*, *Lv3*, *Lv4*, and *Lv5* (lentiviral susceptibility-2, 3, 4, and 5). Here, we review the host antiviral proteins that target lentiviral capsids with a focus on the more poorly understood phenomena that suggest there are additional host antiviral strategies yet to be discovered. We will describe the general phenotypes of each restriction event, discuss the responsible host elements where they are known and summarize the current understanding of events that have not been fully described. Finally, we discuss how new tools could be used to identify unknown blocks to lentiviral infection.

## KNOWN CAPSID-DEPENDENT RESTRICTION FACTORS

### *Fv1* and *Ref1*/*Lv1*/TRIM5α

The first described CA-dependent block to retroviral infection was named Friend virus susceptibility-1 (*Fv1*) (in relation to Friend murine leukemia virus), which inhibited one type of murine leukemia virus (MLV), N-tropic MLV (N-MLV), but not another variant of MLV, B-tropic MLV (Table 1). This block was initially observed in different inbred mouse lines that displayed differential susceptibility to Friend MLV: NIH Swiss mice were permissive to viruses termed N-MLV and not permissive to others termed B-tropic MLV (B-MLV), whereas BALB/c mice were permissive to B-MLV and not permissive to N-MLV (4). The corresponding alleles were termed *Fv1^n^* and *Fv1^b^* (5). Cells derived from these mice expressing the respective alleles were similarly permissive or non-permissive to MLV strains (5). The inhibition was found to occur after reverse transcription and before integration (5). In the mid-1990s, the responsible gene for the non-permissive phenotype was identified (6, 7). Based on sequence homology to the endogenous retroviruses, it is proposed that *Fv1* arose from an integration of an ancient rodent endogenous retrovirus in which the *gag* gene coding sequence remains intact (6). The block occurs via direct interaction with the viral capsid, suggesting interference with capsid integrity and uncoating (8–10). The essential difference between restricted and non-restricted MLV strains is mapped to the CA protein of these viruses (4). Specifically, differential capsid susceptibility to *Fv1* between N-MLV and B-MLV was eventually attributed to a single amino acid change at position 110 in CA (11). *Fv1* activity is also seen in outbred mice, where both gene duplication and amino acid changes confer specificity to restrict different retroviruses (12–15).

**TABLE 1:** Capsid-mediated lentiviral restrictions

Restriction	Susceptible virus(es)	Characteristics
*Fv1^a^*	MLV	Susceptibility determined by CA position 110 Block after reverse transcription Saturable
*Lv1*/*Ref1*/TRIM5α^*b*^	HIV-1	Susceptibility depends on CA identity and host protein identity (SPRY domain) Block before reverse transcription Saturable TRIM5α and other factors involved in sensing of lentiviral capsids
MxB^*c*^	HIV-1	Susceptibility depends on CA identity Block after reverse transcription, before nuclear import
TRIM-CypA and TRIM34	HIV, HIV CA mutants, and SIVs	Susceptibility depends on CA identity Block before reverse transcription
*Lv2^d^*	Certain HIV-2 isolates, HIV-1 CA mutants	Susceptibility determined by both *gag* and *env* HIV-1 CA determinants: P38A, N74D, G89V, and G94D HIV-2 CA determinant: I73V Not saturable Reverse transcription products accumulate more slowly
*Lv3^e^*	HIV-1	Susceptibility determined by *gag*, *env* and entry mechanism/co-receptor utilization Not saturable Block after reverse transcription
*Lv4^f^*	SIV_SMM_/SIV_MAC_/HIV-2 lineage	Susceptibility determined by capsid Occurs in lymphocytes but not epithelial cells Block after reverse transcription
*Lv5^g^*	HIV-1	Present in marmoset primary lymphocytes Block before reverse transcription
Megabat and mouse cell block	HIV-1	Capsid-dependent Block at or before nuclear import

^*a*^ Friend virus susceptibility-1.

^*b*^ Ref1: restriction factor 1; Lv1: lentiviral susceptibility-1.

^*c*^ MxB: Mx Dynamin-like GTPase 2 (Mx2).

^*d*^ Lv2: lentiviral susceptibility-2.

^*e*^ Lv3: lentiviral susceptibility-3.

^*f*^ Lv4: lentiviral susceptibility-4.

^*g*^ Lv5: lentiviral susceptibility-5.

**Fig 1:**
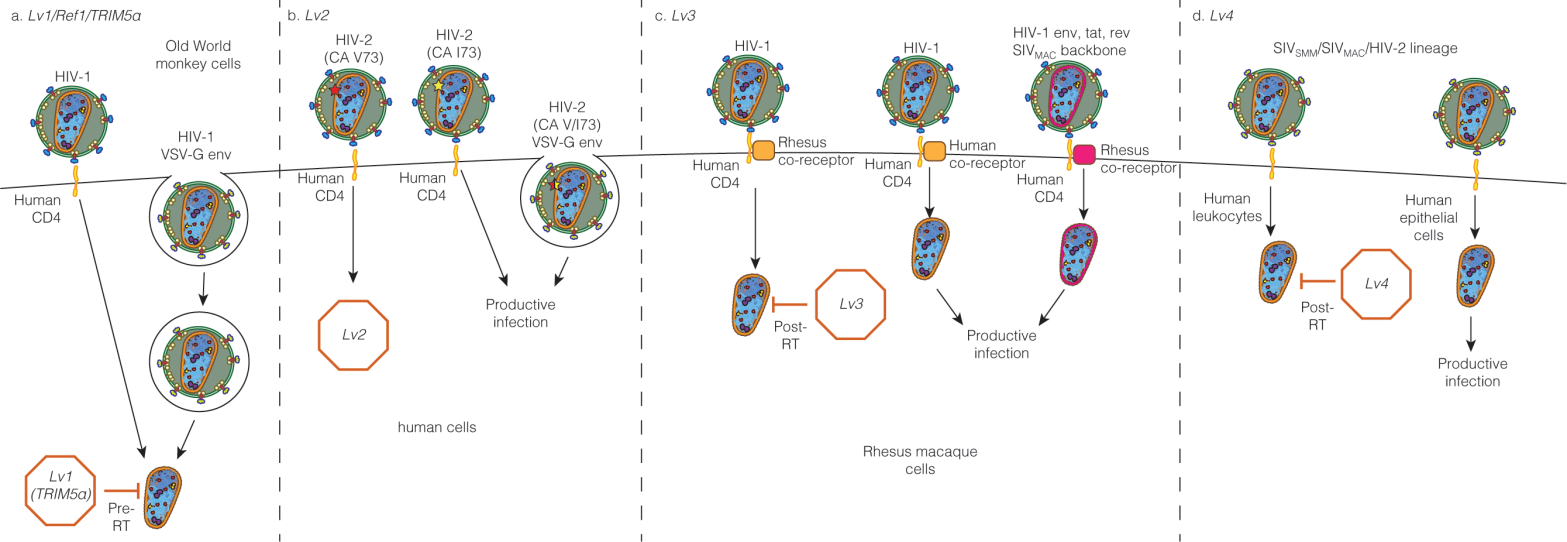
Lentiviral restrictions targeting capsid. (a) *Lv1*/*Ref1/*TRIM5α: HIV entering through either HIV envelope or the VSV-G envelope is restricted in Old World monkey cells by TRIM5α. A similar block is mediated by TRIM-CypA for CypA-binding lentiviruses and by TRIM34 for some HIV capsid mutants and primate lentiviral capsids. MxB inhibits at or before nuclear import. (b) *Lv2*: HIV-2 viruses entering through specific HIV envelopes are restricted in some human cells at a step before completion of reverse transcription. (c) *Lv3*: the *Lv3* block inhibits HIV-1 viruses that enter via a non-human co-receptor at a step after reverse transcription in a rhesus macaque tumor cell line. (d) *Lv4*: Old World monkey (SIV_MAC_ and SIV_SMM_) and HIV-2 capsids are inhibited in human immune cells by a block that restricts infection after reverse transcription. Adapted from Janet Iwasa (26), Creative Commons Attribution-NonCommercial-ShareAlike 4.0 International License.

Subsequent to the discovery of the gene responsible for the *Fv1* block, a block to infection of HIV-1 and MLV in primate cell lines was described with significant similarities to *Fv1* (16–19). Specifically, it was known that some human cell lines could restrict N-MLV but not B-MLV thereby implicating capsid in susceptibility to this restriction (20). Furthermore, HIV-1 was known to be restricted in Old World monkeys by an element that acted on capsid (18, 21, 22). However, this block toHIV-1 was distinct from *Fv1* in that the block to virus replication occurred before, rather than after, reverse transcription (17, 23, 24). Furthermore, a direct human ortholog of the co-opted murine *gag* retroviral gene identified as *Fv1* in mouse cells does not exist in the human genome (20). Therefore, this block in human cells was named restriction factor 1 (*Ref1*) to distinguish it from the mouse cell-specific *Fv1* phenotype (20) (Table 1; Fig. 1a). In addition to *Ref1*, a similar phenotype was observed in African green monkey cells, which could broadly inhibit N-MLV, HIV-1, some Simian immunodeficiency viruses (SIVs) and an even more distantly related lentivirus, equine infectious anemia virus (EIAV) (25). This block in Old World monkey cells was termed lentiviral susceptibility-1 (*Lv1*) (21).

The *Lv1* restriction of HIV-1 in many Old World monkey cells was shown to be mediated by a factor that acted on CA subunits in the viral core (18, 21, 22). Tripartite motif 5 (TRIM5, specifically its alpha isoform TRIM5α) was identified as the factor that inhibits HIV-1 in rhesus macaque cells through expression of a rhesus macaque cDNA library in human cells and a subsequent screen for clones that were resistant to HIV-1 infection (27) (Fig. 1a). By knockdown and overexpression studies, TRIM5α was definitively identified as the host factor underlying both the *Ref1* restriction of N-MLV in human cells and the lentivirus-specific *Lv1* block to HIV-1, some SIVs and EIAV in African green monkey cells (27–32). In other words, *Lv1* was recognized to be a species-specific variant of *Ref1* (29). Therefore, this discovery of TRIM5α restriction accounts for both the *Ref1* and *Lv1* blocks to infection.

TRIM5α, and TRIM proteins more generally, consist of a common set of N-terminal domains (RING, Bbox, and coiled-coil) and at least one variable C-terminal domain (33). In the case of TRIM5α, the C-terminal domain is a B30.2/SPRY domain that binds to CA and is the major determinant of specificity of capsid restriction (34–36). TRIM5α functions by multimerizing into higher-order structures on the viral capsid, resulting in aberrant uncoating and inhibition of viral replication (33). The restriction activity of human TRIM5α is enhanced in cells stimulated with type I interferon (37, 38) in an immunoproteasome-dependent manner (39). Loss of cyclophilin A (CypA) incorporation into HIV-1 virions *via* mutations in CA results in enhanced TRIM5α restriction (40–42). Restriction by TRIM5α may involve TRIM5α-mediating aberrant or premature uncoating of the capsid (43) or by inducing autophagy via the TRIM5α RING domain (44). Models of the TRIM5α restriction mechanism are not mutually exclusive (33).

Consistent with *Lv1*/*Ref1* phenotypes described before its discovery, TRIM5α’s viral specificity is determined by both the host-encoded *TRIM5*α allele and the specific viral capsid (27, 29, 30, 34–36). Due to amino acid differences in the SPRY domains, TRIM5α from humans is a relatively poor restrictor of HIV-1 (2- to 5-fold restriction) whereas rhesus macaque TRIM5α is a potent (100-fold) restrictor of HIV-1 (27, 34). There are a number of individual amino acid residues in CA that have been found to alter TRIM5α susceptibility, including but not limited to P38A (45), G94D (46), A88 (47) and P90A (40, 42).

Although *Fv1* is not a *TRIM* gene and is encoded in an entirely different locus than *TRIM5* (7), the *Lv1*/*Ref1*/TRIM5α block shares several similarities to *Fv1*. These similarities include the following: that viral sensitivity to these blocks is determined by CA amino acid 110 in MLV (11, 28), that the restriction phenotype is saturable (27, 48–50), that the gene is rapidly evolving (34, 51) and that despite no sequence homology there is a similar domain architecture to these proteins that features an N-terminal coiled-coil motif involved in multimerization and a C-terminal domain involved in capsid recognition and binding (13, 33, 52–54).

### TRIM-CypA: a block to CypA-binding lentiviruses

CypA is a host protein with a number of putative functions in the lentiviral life cycle (55). CypA can be bound and incorporated into the virion of some but not all lentiviruses via an approximately eight amino acid-long loop in CA (56–58). Not all lentiviruses bind CypA: for example, HIV-1/SIV_CPZ_ (SIV infecting chimpanzees) lineage viruses do bind CypA, while the HIV-2/SIV_SMM_/SIV_MAC_ (SIVs infecting sooty mangabeys and rhesus macaques, respectively) lineage viruses do not (57, 58). More broadly, CypA is bound by CA of some non-primate lentiviruses (59). CypA binding is important for replication in CypA-binding lentiviruses in the context of restriction. For example, treatment with cyclosporine A (CsA), a CypA inhibitor, results in a block to infection that occurs at the step of reverse transcription (60, 61). Furthermore, the loss of CypA binding in T cells and other immune cell lines decreases replication of HIV-1 (40, 50, 61–65).

Although CypA generally has a positive effect on HIV infection, CypA also contributes to antiviral defense through host cell co-option of the capsid-binding properties of CypA (2, 55, 66). In some primate species, CypA is found as a fusion protein with the N-terminal domains of the restriction factor TRIM5α (67, 68). Of particular relevance to primate lentivirus biology, in some New World monkeys and in primates of the macaque lineage, a retrotransposon-mediated CypA insertion at the C-terminal end of TRIM5α replaces the capsid-binding SPRY domain of canonical TRIM5α (31, 33, 67–73). A TRIM-CypA fusion was found to restrict CypA-binding lentiviruses when attempts to restore CypA function after knockdown of CypA was unsuccessful; northern blotting mapped this unexpected phenotype to restriction mediated by a TRIM-CypA fusion in owl monkey cells (68). Similar to canonical CypA, TRIM-CypA binds some lentiviral capsids via the CypA-binding domain (33, 74). Therefore, the capsid-binding ability of CypA can replace the capsid-binding function of the TRIM5α SPRY domain, while N-terminal domains remain intact and maintain their multimerization functions (75, 76). Similar to TRIM5α, TRIM-CypA blocks lentiviral replication prior to completion of reverse transcription (69, 77). TRIM-CypA has been shown to be a restriction factor of lentiviruses by both knockdown and overexpression studies (67–69). Therefore, unlike the important function of canonical CypA in mediating efficient lentiviral infection, TRIM-CypA acts as an antiviral restriction factor (Table 1).

The antiviral TRIM-CypA phenotype was first observed in owl monkey cells (16, 68). Remarkably, this TRIM-CypA fusion has arisen independently numerous times across vertebrate evolution in, for example, New World monkeys (68), the Asian macaque lineage (69, 71, 78–80), ray-finned fishes (81), shrews (82) and rodents (83). This may reflect the persistent intrusion of CypA-binding viruses throughout evolutionary history and repeated selection for this form of an antiviral fusion protein.

### TRIM34

In addition to *TRIM5*, primate genomes encode approximately 70–100 other *TRIM* genes (84). *TRIM5* itself exists in a gene locus with three paralogous *TRIM* family members *(TRIM34*, *TRIM6*, and *TRIM22)*; these are the most closely related *TRIM* genes to *TRIM5* in the human genome (85, 86). TRIM34 was first identified as ring finger 21 in a screen to identify novel RING domain-containing proteins in the human genome (87). Similar to TRIM5, TRIM34 is broadly expressed across many cell types and is upregulated by type I interferons such as Interferon alpha (IFN-α) (87).

TRIM34 antiviral function has recently been described. An HIV-1 CA mutant virus (N74D) was shown to be more sensitive to IFN-α-mediated blocks relative to wild-type HIV-1, suggesting the presence of one or more unknown restriction factors (62). The N74D CA mutant virus does not bind to the host protein CPSF6 (88, 89). TRIM34 was identified as a restriction factor of this HIV-1 CA mutant (CA N74D) using an HIV-specificCRISPR (clustered regularly interspaced short palindromic repeats) screening approach (38, 41). In earlier studies, TRIM34 was not shown to have any anti-retroviral activity against HIV-1, but this did not include testing of what are now known to be TRIM34-susceptible HIV viruses (76). TRIM34 restriction has been demonstrated through knockout, knockdown and overexpression studies and has a 2-fold to 10-fold effect on virus replication (41, 90). Susceptibility to TRIM34 restriction and CPSF6 binding appear to be independent of one another as other mutations that abrogate CPSF6 binding are not sensitive to TRIM34 restriction (41). TRIM34 restricts lentiviruses prior to reverse transcription and does not require Interferon for activity (41). TRIM34 restricts other primate lentiviral capsids including SIV_MAC_ and SIV_AGM-TAN_ (an SIV originating from tantalus monkeys) (41, 90) (Table 1). Of note, TRIM34 does not function independently as TRIM5α is necessary for TRIM34-mediated restriction (41, 90). TRIM34 may multimerize with TRIM5α in order to restrict a subset of lentiviruses (41, 90). Possible models of how TRIM5α may contribute to TRIM34 activity include: acting as an effector molecule through its RING domain, providing structural support via its Bbox or coiled-coil domains or modifying binding specificity through its B30.2/SPRY domain (41, 90).

### MxB

Myxovirus resistance protein B (MxB; also known as human Mx2) also restricts lentiviral capsids (Table 1). *Mx* genes are conserved across vertebrates and have expanded via ancient gene duplication and conversion events (91). Mx proteins comprise a dynamin-like large GTPase domain followed by a helical region and a hinge-like bundle-signaling element (92). Humans encode two Mx proteins with known antiviral function: MxA, the human ortholog of mouse Mx1 and Mx2, and MxB (93). Human Mx proteins can restrict a broad range of viruses, including but not limited to influenza virus (94, 95), Thogoto virus (96, 97), vesicular stomatitis virus (95, 98), human parainfluenza virus (99), herpesviruses (100, 101), and hepatitis B virus (102).

Human MxB was shown by overexpression and knockdown studies to inhibit HIV-1 replication after MxB was identified as a candidate gene expressed upon Interferon-mediated induction in non-permissive cells (103–105). MxB interferes with HIV nuclear import and perhaps subsequent proviral integration (103–106). MxB localizes at the nuclear pore complex and depends on the presence of a nuclear localization sequence for its activity (106, 107). Furthermore, MxB restriction depends on which nuclear pore proteins are utilized for nuclear entry and is linked to GTPase activity (106–108). Mx proteins form dimers and higher-order oligomers (92, 109). Dimerization is required for MxB antiviral activity against HIV-1 (110, 111). Recent experiments suggest that MxB may act as a decoy, luring HIV cores away from nuclear pores and thus impeding nuclear entry (112).

As with TRIM5α, restriction of HIV-1 by MxB is dependent on capsid sequence as point mutations in CA can markedly alter susceptibility of HIV capsids to MxB restriction (103–106, 113, 114). Most, if not all, CA mutations tested to date appear to reduce MxB sensitivity rather than enhance it. That restriction of HIV-1 by MxB can be abrogated or altered due to sequence differences in CA highlights the possibility of a direct interface of MxB with lentiviral capsids (115). However, sequence differences in CA could also have indirect effects on MxB restriction. For example, a change in susceptibility to TRIM5α could impact how much MxB restriction is observed as TRIM5α also interacts with capsid and could mask effects of MxB. In addition, CA sequence impacts host factor interactions, including those with CypA, and such differential interactions may impact restriction factor susceptibility (107, 108). Interaction of HIV capsids with host CypA is important for MxB restriction as disruption of CypA binding to CA also results in abrogation of MxB restriction (104, 105, 107, 108).

### Capsid-binding factors as innate immune sensors

In addition to interacting with viral proteins to directly inhibit the viral life cycle, host proteins can sense lentiviral capsids and activate innate immune signaling as a mechanism of indirect inhibition of viral replication. For example, in addition to direct viral inhibition, TRIM5α possesses an innate immune detection and signaling function in the presence of viral infection (116–118). After CA recognition by the SPRY domain, the TRIM5α RING domain can act as an E3 ubiquitin ligase which generates K63-linked ubiquitin chains, thereby activating innate immune responses (116, 119, 120). Lentiviral capsids are differentially sensitive to detection by TRIM5α in human cells (121).

Innate immune induction also occurs when the host protein non-POU domain containing octamer binding (NONO) binds HIV-2 CA inside the nucleus and complexes with nuclear cGAS, resulting in cGAS sensing of viral DNA (122). This, in turn, leads to stimulator of interferon response cGAMP activator 1 (STING) activation and induction of an antiviral gene program (122–124). NONO has evolved under negative selection and recognizes a conserved epitope of the CA protein (122). Another host factor that has been proposed to be implicated in lentiviral capsid sensing is polyglutamine-binding protein 1 (PQBP1) (125). In contrast to NONO, which binds to CA inside the nucleus, PQBP1 is proposed to bind to intact viral cores in the cytoplasm (125–127). After the initiation of capsid disassembly and reverse transcription, PQBP1 recruits cGAS to the capsid, allowing it to sense viral DNA and induce innate immune activation (125, 126). Taken together, these factors contribute indirectly but significantly to the capsid-dependent blocks to infection in infected host cells.

## UNKNOWN CAPSID-DEPENDENT RESTRICTIONS

While TRIM5α was identified as the cellular component responsible for the *Lv1*/*Ref1* restriction, there exist other known blocks to lentiviral infection that depend on capsid that remain poorly understood and for which the genes responsible have not been identified. We will discuss a set of these, termed *Lv2*, *Lv3*, *Lv4*, and *Lv5*, as well as some additional, less well-characterized blocks.

### *Lv2*: an entry and capsid-dependent block to HIV-2 infection

A block to lentiviral infection discovered after *Lv1* was named *Lv2* (128, 129) (Table 1; Fig. 1b). *Lv2* was first described as a block to infection by a primary HIV-2 isolate called molecular clone restricted (MCR) (128). *Lv2* is a block to infection by MCR in primary macrophages, immortalized fibroblasts, and some immortalized epithelial cell lines but not in peripheral blood mononuclear cells (PBMCs), immortalized T cells and other immortalized epithelial cell lines (130). This is in contrast to a closely related T cell line-adapted HIV-2 isolate called molecular clone non-restricted (MCN), which replicates well in all cell lines tested (130). The *Lv2* block results in roughly 10- to 100-fold less infectivity for MCR compared to MCN, depending on the cell type (130). MCR HIV-2 is hypothesized to be restricted by a host factor that does not target MCN HIV-2. Several correlates to restriction for MCR and MCN have been identified. The restricted MCR HIV-2 and the unrestricted MCN HIV-2 differ in *gag* and *env* sequences, with both genes playing a key role in determining *Lv2* sensitivity (128, 131). Specifically, in terms of the role of capsid, a single amino acid at position 73 in CA (Gag 207) confers sensitivity and resistance to *Lv2* (128).

As *Lv2* restriction shows a dependence on capsid, TRIM5α was hypothesized to perhaps play a role in this restriction. However, several lines of evidence support that *Lv2* restriction is distinct from TRIM5α. First, TRIM5α is saturable by pre-treatment with N-MLV, meaning that addition of a sufficient saturating amount N-MLV will prevent TRIM5α from being able to restrict other capsids (128). *Lv2* is not saturable by addition of a different TRIM5α-restricted retrovirus, suggesting that *Lv2* restriction is independent of TRIM5α (128). Second, the *Lv2* restriction can be overcome by VSV-G pseudotyping, which bypasses receptor-mediated fusion and instead causes virus entry via an endocytic pathway (132), whereas TRIM5α restriction is not affected by VSV-G pseudotyping (128, 130). Finally, the I73V CA mutant (Gag I207V) is not sensitive to *Lv2* restriction but is susceptible to restriction by TRIM5α (131, 133).

One notable aspect of the *Lv2* block is that, in addition to being dependent on capsid, it is also entry pathway-dependent with post-fusion trafficking events also playing a role. VSV-G pseudotyping was shown to rescue MCR HIV-2 as well as numerous other restricted HIV-1 and HIV-2 strains from *Lv2*, supporting the entry dependence of the Lv2 block more broadly (128, 134). Therefore, *Lv2* restriction may include a host factor that acts specifically after receptor-mediated entry and that can be bypassed by viruses that enter via alternative routes such as endocytosis. An *Lv2*-sensitive capsid might escape restriction through utilization of an entry pathway in which it does not subsequently encounter *Lv2* due to differential compartmentalization or trafficking pathways.

At least one gene candidate to explain *Lv2* restriction has been identified. An siRNA screen to identify the host factors responsible for the *Lv2* block against MCR HIV-2 identified RNA-associate early-stage antiviral factor (REAF) (also known as regulation of nuclear pre-MRNA domain 2) as *Lv2* (135). REAF was shown by knockdown to be implicated in restriction of MCR HIV-2 (135). When tested against an HIV-1 virus, knockdown of REAF relieved restriction about 50-fold, and overexpression of REAF resulted in about a 3-fold decrease in infectivity, supporting a model in which REAF can also restrict some HIV-1 viruses (135). Several single amino acid mutations in CA were found to be critical for REAF-mediated inhibition of HIV-1: in particular, the HIV-1 CA mutations P38A, N74D, G89V, and G94D increased sensitivity to *Lv2* (136). Residue 74 in CA is important for CPSF6 binding (137) and more recently has been implicated in TRIM34 restriction (41). G89V and G94D are located in the CypA-binding loop in CA (138, 139). Notably, although CA I73V (Gag I207V) is an important determinant of sensitivity for Lv2 restriction of HIV-2, the equivalent residue in HIV-1 is not an important determinant of REAF susceptibility (136). In fact, the P38A, N74D, G89V, and G94D mutations in HIV-1 CA are stronger determinants of susceptibility to REAF than CA 73 (Gag 207) is for restriction of HIV-2 (136). This could suggest that the primary determinants of susceptibility to REAF differ between HIV-1 and HIV-2 due to other differences in CA or even other viral proteins. Alternatively, this could indicate that REAF is only one component of *Lv2* and that *Lv2* susceptibility depends on more than one host factor. For example, while REAF might be sufficient on its own to restrict HIV-2, it might require other host components to restrict HIV-1. The most recent work on REAF restriction suggests that HIV-1 can use the accessory protein Vpr to overcome REAF-mediated restriction (140). This raises the question of whether HIV-2 can also use Vpr to antagonize REAF. While some aspects of the *Lv2* block may be explained by REAF activity, we believe that the available evidence is not sufficient to support REAF as the major or sole component of *Lv2* restriction.

### *Lv4*: a block to Old World monkey lentiviral capsids in human leukocytes

*Lv2* is characterized by differential susceptibility to restriction of HIV-2 across human cell types. In contrast, *Lv4* is a block to lentiviral infection that is defined by differential inhibition of HIV and SIV strains in specific types of human cells but not in others (141) (Table 1; Fig. 1d). Infection by SIV_MAC_ is not efficiently blocked in some human epithelial cell lines and this is correlated with a lack of restriction of SIV_MAC_ by human TRIM5α (17, 19, 21, 27, 41, 142). However, SIV_MAC_, as well as SIV_SMM_ (SIV from sooty mangabeys) and HIV-2, are less infectious than HIV-1_NL4-3_ in human leukocytes such as bulk PBMCs, human B cells, T cells, myeloid cells, and dendritic cells (16, 141). For example, HIV-1 is 50 times more efficient than SIV_MAC_239 and 10 times more efficient than HIV-2_ROD_ in infecting these cell types (141). The differential restriction of SIV_MAC_ viruses across human cell types suggests the potential presence of a cell type-specific restriction activity or lack of a required factor for SIV_MAC_ replication in these immune cells (141). This block against SIV_SMM_/SIV_MAC_/HIV-2 lineage viruses in some human cells is termed *Lv4* (141).

Substitution of HIV-1 CA with SIV_MAC_, SIV_SMM_, or HIV-2 CA is sufficient to reduce infectivity of the CA chimeric viruses, supporting the hypothesis that the *Lv4* block targets capsid (141). To ask if the *Lv4* restriction is due to a positive or negative factor, heterokaryon cell fusions were generated from HeLa cells (epithelial cell line, permissive) and Jurkat cells (T-cell leukemia cell line, non-permissive) (141). These heterokaryons are restrictive similar to Jurkat cells alone in that they cannot be infected by SIV_MAC_ CA-containing viruses, supporting the hypothesis that there exists a dominant antiviral factor expressed in immune cell types that restricts SIV_SMM_/SIV_MAC_/HIV-2 lineage viruses but does not restrict HIV-1 (141). Like TRIM5α, but unlike *Lv2*, *Lv4* is not affected by pseudotyping and is therefore entry pathway independent (16, 141). In contrast to TRIM5α, the block to infection occurs after reverse transcription and nuclear import (141). A similar post-reverse transcription, pre-integration block has also been observed for some CA mutant viruses that escape from cytotoxic T lymphocytes (143). TRIM5α blocks HIV replication at a later step (post-reverse transcription) if cells are treated with a proteasome inhibitor such as MG132 (144). A similar effect is observed if mutations are introduced into the RING E3 ubiquitin ligase domain of TRIM5α (145). One possibility is that the factor(s) resulting in the *Lv4* block is/are functioning similar to an E3 ligase activity-deficient TRIM5α perhaps by binding to capsids and blocking successful integration. In summary, *Lv4* is likely one or more human factors expressed in some immune cell lines but not epithelial cells that inhibit SIV_SMM_/SIV_MAC_/HIV-2 lineage viruses.

## OTHER POST-ENTRY RESTRICTIONS IN NON-HUMAN CELLS

### *Lv3*: a block against HIV-1 infection in a rhesus macaque cell line

There are additional post-entry restrictions to HIV-1 in non-human cells that appear similar to TRIM5α but are independent of TRIM5α in each case. For example, CMMT/CD4 cells, rhesus mammary gland tumor cells engineered to express human CD4, are susceptible to efficient infection by SIV_SMM_, SIV_MAC_, and SIV_AGM_ (SIV originating from one of the African green monkey species) (146) and HIV-2 (147) but are not susceptible to infection by HIV-1 (147) (Table 1; Fig. 1c). Interestingly, in these CMMT/CD4 cells, some HIV-1 isolates are blocked before initiation of reverse transcription, while others are blocked after reverse transcription (148). The block before reverse transcription is now known to be due to TRIM5α (149). However, the block occurring after reverse transcription supports the existence of another factor in these rhesus macaque cells that also restricts HIV-1. This block is termed *Lv3* (149) (Table 1; Fig. 1c). In CMMT/CD4 where expression of TRIM5α has been knocked-down, this block results in about 20 times less infectivity of HIV-1 compared to infectivity in TRIM5α knockdown HeLa/CD4 cells (149). Further evidence supports the idea that *Lv3* restriction is distinct from TRIM5α. First, *Lv3* restriction is not saturable (149). Second, while VSV-G pseudotyping does not rescue viruses from TRIM5α restriction, VSV-G pseudotyping enables escape from the *Lv3* block occurring after reverse transcription (149). Thus, like *Lv2*, the pathway of entry plays a role in determining sensitivity of HIV to *Lv3*.

Further highlighting the importance of entry pathway to *Lv3* restriction, productive infection of these rhesus macaque CMMT/CD4 cells by restricted HIV-1 can be rescued by overexpression of the human co-receptor (CXCR4 or CCR5) (148). Therefore, entry of HIV via the human co-receptor allows escape from *Lv3*-mediated restriction. Chimeric viruses made up of both SIV and HIV sequences (“SHIVs”) that consist of predominantly SIV_MAC_ sequence but with a backbone containing *env*, *tat*, and *rev* from HIV-1 also productively infect these *Lv3*-encoding rhesus macaque cells (148). This indicates that HIV-1 *env* is sufficient for the virus to bind and enter via a macaque co-receptor, but other viral components determine sensitivity to the *Lv3* block (148). One possibility is that differences in signaling after receptor-mediated entry could play a role. For example, the tyrosine kinase Lck has been shown to be activated upon env engagement with CD4 (150); it may be that signaling downstream of receptor engagement could be important for *Lv3* restriction. Overall, these findings support a model in which productive infection involves both the envelope and perhaps CA, although other viral components could also be important for *Lv3*.

### *Lv5*: a block to infection of marmoset primary cells

HIV-1 infection of primary peripheral blood lymphocytes from common marmosets, a type of New World monkey, is also blocked by one or more restriction factors (151) (Table 1). Compared to human peripheral blood lymphocytes, marmoset peripheral blood lymphocytes are about 10 times less permissive to HIV-1 (151). This dominant post-entry phenotype, called *Lv5*, acts before reverse transcription and is not influenced by the mode of viral entry (151). *Lv5* restriction does not appear to be explained by TRIM5α activity, as TRIM5α cloned from marmoset cells does not inhibit HIV-1 (151). Furthermore, TRIM-CypA does not contribute to the *Lv5* block as this gene fusion has not been identified in marmoset cells (151). It is not known if capsid is directly involved in the *Lv5* restriction, although a separate earlier block to infection in marmoset cells is influenced by mutations in HIV-1 CA, including N74D (151).

### Restrictions to HIV-1 in megabat and mouse cells

Similar to *Lv5*, some species of megabats appear to encode a post-entry, dominant restriction factor to HIV-1 that is CA dependent and blocks at or before nuclear entry (152, 153) (Table 1). This bat restriction is not encoded by an ortholog of any of the known primate restriction factors so far identified in these bat species that act in a CA-dependent manner (152, 153). Likewise, a block to infection in murine T cells occurs after reverse transcription but before integration (154–156). Treatment with CsA, a CypA inhibitor, relieves the pre-integration block in mouse cells (which might implicate an effect of CypA on HIV-1 CA), but even in the presence of CsA, post-integration defects that are independent of CyclinT1, a factor known to be necessary for HIV to infection mouse cells, still remain (157).

## NEW APPROACHES TO CHARACTERIZE BLOCKS TO LENTIVIRAL INFECTION

We have herein described known capsid-binding retroviral restriction factors*—Fv1*, TRIM5α, TRIM-CypA, TRIM34, and MxB—and discussed additional blocks to lentiviral infection that are at least partially capsid-targeting restriction phenotypes that are not fully understood (Table 1). *Lv2* seems to be entry pathway dependent, and sensitivity of lentiviruses to *Lv2* is determined by both capsid and envelope sequence (128, 129). *Lv3* is characterized as a block against HIV-1 in a rhesus macaque mammary tumor cell line and depends on envelope and another viral component that may include CA (147–149). *Lv4* is mediated by a dominant factor in immune cell types and targets capsids from the SIV_SMM_/SIV_MAC_/HIV-2 lineage (141). *Lv5* is mediated by a dominant factor in marmoset primary lymphocytes that acts post-entry and prior to reverse transcription (151). Similarly, sensitivity to an unknown restriction in bat cells is dependent on CA (152, 153).

Genes responsible for these blocks have been uncovered through various approaches over decades. However, as detailed in this review many blocks to lentiviral infection remain unexplained. It is possible that some of the genes responsible for these unknown restriction phenotypes may already have been described as restrictions but not directly connected with these characterized but not yet fully explained blocks to infection. For example, interferon-induced transmembrane proteins (IFITMs) are a family of interferon-stimulated proteins that interfere with viral entry by altering membrane components and/or by altering vesicular trafficking (158). More specifically, IFITMs act by impeding viral entry and localize to endocytic compartments but do not directly inhibit endocytosis (159). IFITMs can inhibit infection by a wide range of viruses including, but not limited to, influenza viruses (160), flaviviruses (160), coronaviruses (161, 162), filoviruses (161), rhabdoviruses (163), alphaviruses (164), and retroviruses, including HIV (159, 165). IFITM2 and IFITM3, in particular, have been shown to block HIV-1 entry (159). Rapamycin, a drug that increases transduction by HIV-based vectors, was shown to enhance transduction through degradation of IFITM3 (166). IFITM3 has also been shown to possess activity against HIV-2, SIV_CPZ_, SIV_MAC_ and SIV_AGM_ (165). Therefore, it may be that some portion of unknown blocks to infection could be at least partially explained by IFITM restriction. The lack of connection of IFITM restriction with these unexplained blocks to infection could be due to one of the major challenges in the study of IFITMs: IFITMs function both after incorporation into virions as well as when expressed in target cells to inhibit incoming virus, making their function more complicated to assess than many other restriction factors (167). Furthermore, similar to the gene duplication and expansion observed for many antiviral gene families, humans encode five IFITMs, at least three of which possess antiviral properties (167, 168). Therefore IFITMs may have redundant function, making experiments to assess their role in a particular restriction phenotype through genetic deletions more technically challenging.

Similar to *IFITM* family members, the *TRIM* gene family is also a good candidate for finding unknown capsid-targeting restrictions. Previously described antiviral functions of some TRIM proteins may explain some unknown blocks to infection. For example, TRIM11 was identified as a restriction factor of HIV-1 in a screen of several dozen TRIMs for antiviral activity and could be involved in the *Lv2* phenotype (169). TRIM11 inhibits HIV viral entry and affects microtubule trafficking, but it is independent of the lysosome and the proteasome, consistent with observations of *Lv2* (169, 170). The TRIM11 block occurs before reverse transcription and results in accelerated uncoating (170, 171). As with *Lv2*, TRIM11 restricts HIV-1 N74D and G94V CA mutant viruses (171); conversely, a G89V CA mutant is insensitive to TRIM11 restriction but restricted by Lv2 (136, 171). Conducting knockout and complementation experiments with TRIM11 could establish whether it is necessary or sufficient for any or all of the *Lv2* block. Similarly, other *TRIM* gene family members are good candidates for capsid-dependent restriction more generally. In addition to potential functional redundancy, a challenge in assessing the role of TRIMs in restriction phenotypes is that TRIM proteins are known to both homomultimerize and heteromultimerize with other TRIMs as a part of their antiviral function. This heteromultimerization could be important for function as is the case for restriction of HIV-1 CA N74D and SIVs by TRIM34 and TRIM5α (41, 86, 90). Functional studies of TRIM restriction are significantly more complicated if there are more than one *TRIM* gene involved in a restriction phenotype but approaches to assess heteromultimeric TRIM restriction should be developed and considered in future work.

There are other significant challenges in identifying genes responsible for unknown blocks to infection. For example, as it is possible that more than one host protein contributes to a given block, a combination of strategies might be needed to identify all components. These factors could be independently acting proteins or together may be required for a given block to infection. While most lentiviral restriction factors that are presently known appear to act alone, some, such as TRIM34, require the presence of another protein for restriction (41). It is possible that this kind of multiprotein cooperation could also occur in other restriction events. Finally, genes encoding these restriction blocks may be shared across multiple *Lv* phenotypes, and this should be considered when assessing the role of different antiviral genes in these blocks.

Technological advances since the initial characterization of these lentiviral restriction phenotypes may permit the identification of some or all of the cellular components responsible for *Lv2*, *Lv3*, *Lv4*, and *Lv5* as well the other restrictions we have discussed and other blocks yet to be identified. For example, CRISPR editing technologies have revolutionized the process of quickly and accurately generating gene knockouts, a particularly powerful method to identify genes required for lentiviral restriction. A CRISPR screening approach could be employed using specific cells and viruses to identify antiviral factors underlying the unknown blocks described herein (38, 172, 173). Screening with genome-wide libraries is useful for unbiased screening. Alternatively, libraries based on known interferon-stimulated genes, TRIM family genes or other genes thought to have antiviral properties could be employed (174, 175). These types of approaches may uncover currently unknown restriction factors in addition to ones that have previously been identified in the literature but have not been demonstrated to play a role in these specific phenotypes. If the antiviral genes in these blocks have redundant functions, these genetic deletion approaches may not be successful. However, other CRISPR-based functional genomics approaches, such as CRISPR activation screens that lead to candidate gene overexpression, could prove fruitful (176).

## CAPSID AS A TARGET FOR HOST RESTRICTION

We note that restriction factors that target the HIV capsid are of interest beyond the blocks to infection we have described here. Compared to other viral epitopes such as *env*, which is known for its mutational resilience and flexibility (177, 178), capsid is highly genetically fragile (179, 180). Proper maintenance of capsid integrity and the occurrence of proper uncoating spatially and temporally are important to a number of steps in the viral life cycle, including reverse transcription and nuclear import (1). Perhaps due to the relative abundance of capsid, its importance to the early steps of the viral life cycle and the lack of expression of viral gene products at this stage of infection, the incoming viral capsid appears to be a key target for host restriction. Identifying and characterizing host capsid-targeting lentiviral restrictions may lead to a better understanding of capsid susceptibilities that could be targeted therapeutically.
